# Virtual hospital-level care—feasibility, acceptability, safety and impact of a pilot Hospital-In-The-Home model for COVID-19 infection

**DOI:** 10.3389/fdgth.2023.1068444

**Published:** 2023-04-05

**Authors:** J. Lawrence, D. Truong, A. Dao, P. A. Bryant

**Affiliations:** ^1^ Electronic Medical Record Team, Royal Children’s Hospital, Melbourne, VIC, Australia; ^2^ Hospital-in-the-Home Department, Royal Children’s Hospital, Melbourne, VIC, Australia; ^3^ Health Services Research Unit, Murdoch Children’s Research Institute, Melbourne, VIC, Australia; ^4^ Department of Paediatrics, University of Melbourne, Parkville, VIC, Australia; ^5^ Radiology Department, Royal Melbourne Hospital, Melbourne, VIC, Australia; ^6^ Infectious Disease Unit, Royal Children’s Hospital, Melbourne, VIC, Australia; ^7^ Clinical Paediatrics Group, Murdoch Children’s Research Institute, Melbourne, Australia

**Keywords:** virtual, paedaitrics, hospital at home, sustainability, teleconsultation, remote monitoring

## Abstract

**Background:**

Hospital-in-the-Home (HITH) delivers hospital level care to patients in the comfort of their own home. Traditionally HITH involves clinicians travelling to patients' homes. We designed and implemented a virtual model of care leveraging a combination of virtual health modalities for children with COVID-19 in response to rising patient numbers, infection risk and pressures on protective equipment. In contrast to other models for COVID-19 infection in Australia at the time, our HITH service catered only for children who were unwell enough to meet criteria for hospitalisation (ie bed-replacement).

**Aims:**

To measure the feasibility, acceptability, safety and impact of a virtual model of care for managing children with COVID-19 infection requiring hospital-level care.

**Methods:**

Retrospective study of a new virtual model of care for all children admitted to the Royal Children's HITH service with COVID-19 infection between 7th October 2021 and 28th April 2022. The model consisted of at least daily video consultations, remote oximetry, symptom tracking, portal messaging and 24 h phone and video support. Patients were eligible if they met a certain level of severity (work of breathing, dehydration, lower oxygen saturations) without requiring intravenous fluids, oxygen support or intensive care. Online surveys were distributed to staff and consumers who experienced the model of care.

**Results:**

331 patients were managed through the virtual HITH program with a mean length of stay of 3.5 days. Of these, 331 (100%) engaged in video consultations, 192 (58%) engaged in the patient portal and completed the symptom tracker a total of 634 times and communicated *via* a total of 783 messages. Consumer satisfaction (*n* = 31) was high (4.7/5) with the most useful aspect of the model rated as video consultation. Clinician satisfaction (*n* = 9) was also high with a net promoter score of 8.9. There were no adverse events at home. Eight children (2.4%) represented to hospital, 7 (2.1%) of whom were readmitted. The impact is represented by a total of 1,312 hospital bed-days saved in the seven-month period (2,249 bed-days per year). In addition, 1,480 home visits (travel time/ protective equipment/ infection risk) were avoided.

**Conclusion:**

A virtual HITH program for COVID-19 in children is feasible, acceptable and safe and has a substantial impact on bed-days saved and nursing travel time. The implications for management of other acute respiratory viral illnesses that contribute to hospital bed pressure during winter months is immense. Virtual HITH is likely to be a key enabler of a sustainable healthcare system.

## Introduction

Without an intense effort to improve hospital sustainability, healthcare system expenditure is fast becoming unsustainable (1). Hospital-In-The-Home (HITH) offers a scalable model of care that offers a solution to bed blockage and hospital overcrowding. HITH provides acute hospital level care *via* clinical staff travelling to patients' homes to deliver the required care intervention, including intravenous antibiotics, wound care or chemotherapy (2). Not only does this reduce pressure on physical hospital beds, it also offers psychological benefits and saves costs for both the healthcare system and families (3, 4). A meta-analysis comparing HITH to in-hospital care for adults showed reduced mortality, readmission rates and cost whilst achieving higher patient satisfaction (2). In paediatric HITH, studies mostly focus on IV antibiotic administration but equally demonstrate that HITH is safe and cost-effective (4, 5). The arrival of the COVID-19 pandemic in 2020 resulted in an exponential rise in COVID-19 cases across the state and an uncertain peak demand for hospital-level care (6) the role for HITH to support patients at home became critical to avoid hospital overcrowding and bed block.

The HITH program at our institution had some early experience managing infants with bronchiolitis in the home, with nurses visiting daily for respiratory and hydration reviews. With early COVID-19 admissions to our program, we replicated this model of care with in-person daily visits to assess respiratory and hydration status. Nurses used personal protective equipment (PPE) on the road with procedures developed to don and doff safely in the car. With an exponential rise in cases and an uncertain peak demand, a new model of care was conceived that would provide the same level of support to children with COVID-19 infection virtually. This was enabled by a dramatic culture shift in the ambulatory space to video consultations replacing face-to-face visits, and a growing appetite amongst consumers for virtual models of care (7).

There has been an explosion of virtual emergency services, ambulatory video visits and in many centres virtual ward rounds in paediatric health care since the beginning of the pandemic (8–11). However despite this growth in virtual care models around the globe, much knowledge sharing has been informal with the speed of implementation inhibiting robust evaluation. Whilst consumer experience of virtual care models has gained substantial attention, literature on the impact of virtual models on clinical outcomes, equity, safety and cost-effectiveness remains sparse. Transitioning to a virtual model offers the ability to scale and offer quality clinical care to more patients with the same clinical resource through reduction of travel time and ability to prioritise patients, collect relevant information prior to the consultation and avoid time spent trying to contact one another *via* phone.

**Figure 1:**
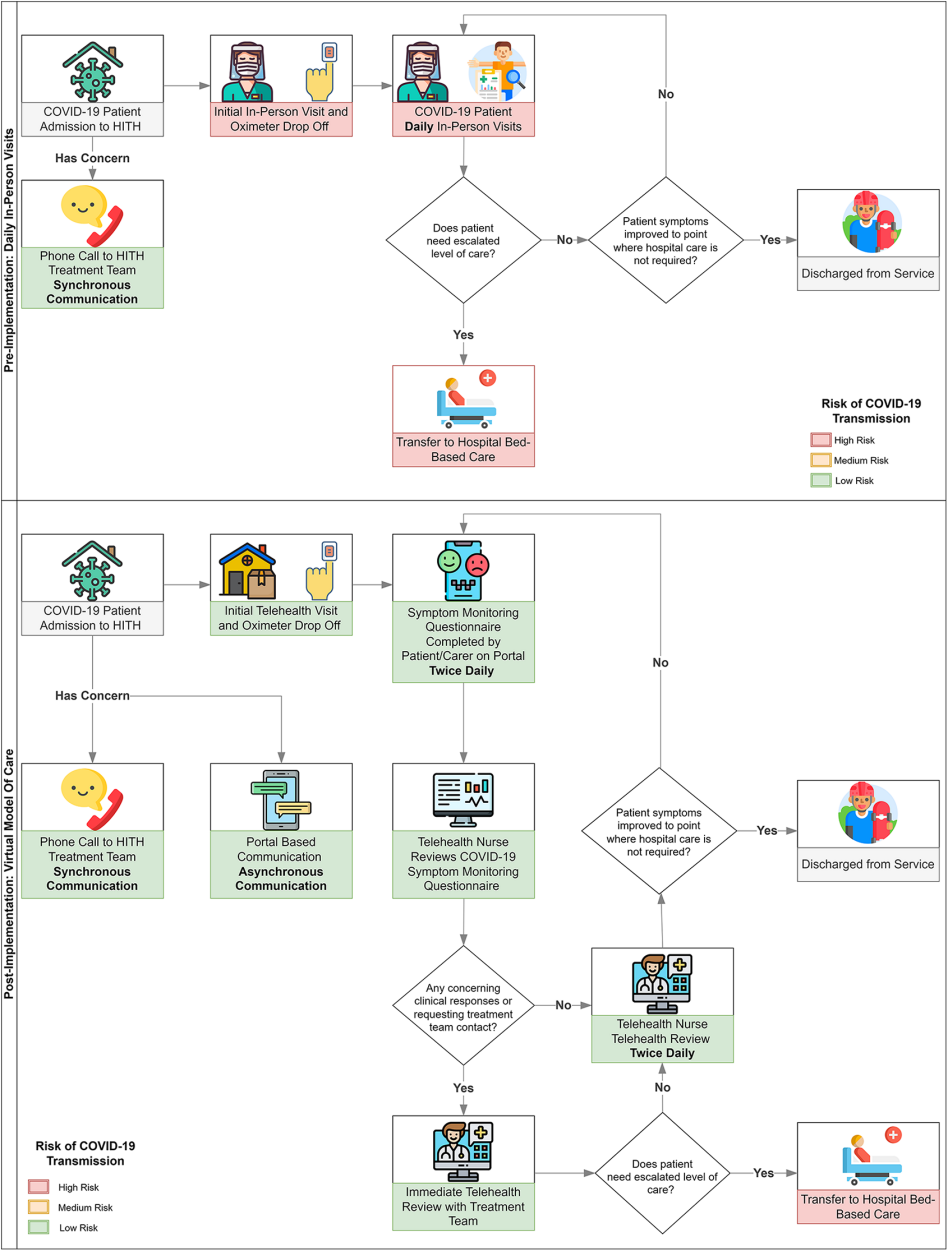
Pre and Post Virtual HITH COVID-19 Model.

Criteria for admission to HITH at our institution required a level of severity that warranted observation in hospital. This is a key differentiator from many of the models rapidly implemented across Australia, which offered observation for milder levels of illness. The model included one or more video consultations per day augmented by oximetry in the home for assessment of vital signs. In addition, twice daily, patients were asked to complete a symptom tracker through the hospital electronic medical record (EMR) patient-facing portal. Results from the symptom tracker fed into a clinical dashboard allowing clinicians to easily recognise patients who were deteriorating and required further follow-up. The portal also enabled patients and clinicians to communicate with each other through asynchronous communication and avoid multiple phone calls at a time where multiple services were attempting to contact families (department of health, contact tracing, adult HITH services) (Figure 1).

We aimed to assess the success of this virtual care model, to determine its implications for the future care of children at home for COVID-19 and other illnesses.

## Aims

To measure the feasibility, acceptability, safety and impact of managing patients with COVID-19 infection requiring virtual HITH care.

## Methods

The Royal Children's Hospital (RCH) HITH is the largest paediatric HITH service in Australia. Children (0–18 years) admitted to the HITH service with COVID-19 infection between 7th October and 28th April 2022 were included in the study. All children were managed with a virtual model of care (Figure 1) that included video consultation at least daily (and as required), twice daily symptom tracking (questionnaire) through the portal, remote oximetry to support both video consultations and symptom tracking, and 24-hour support *via* phone with ability to escalate to video consultation or in person attendance to hospital. The symptom tracking questionnaire asked patients to report on the absence, presence and severity of common symptoms associated with COVID-19 infection as well as their general level of concern. Oximetry results and temperature were also entered through the symptom tracker twice daily. Algorithms were built to alert clinicians if symptoms or vital signs breached certain thresholds, otherwise clinician dashboards allowed clinicians to easily visualise which patients were improving, stable or deteriorating to allow prioritisation of patient reviews. The portal also enabled asynchronous messaging enabling consumers and staff to communicate without having to be available at the same time.

We developed an evaluation framework based on existing frameworks but adapted for our local context (Table 1) (12–15).

**Table 1:** RCH virtual care evaluation framework.

Evaluation Component	Definition	Metrics
Feasibility	The ability for clinicians and patients to interact and engage with the model of care.	• Number of video consultations successfully completed. • Number of patients who could activate a portal account. • Number of symptom trackers completed. • Number of portal messages sent. • Number of patients successfully using remote monitoring.
Acceptability	Whether patients/families and clinicians found the model fit for purpose and met their needs.	• Consumer satisfaction—sense of safety, impact on stress, perceived timeliness of care, overall satisfaction. • Clinician satisfaction—Net Promotor Score (would they recommend this model?).
Impact	Measurable outcomes on hospital efficiency and the environment.	• In-hospital bed-days saved. • Number of patients rostered onto a nurse's shift. • Time saved in travel to and from the patient's home. • Environmental impact—kg CO2 saved.
Safety	Whether the new virtual model led to adverse outcomes.	• Number of adverse outcomes. • Number of emergency reviews required. • Number of patients who required escalation to intensive care.

Feasibility: The ability for clinicians, patients and their families to engage in the tools and functionality available was measured through the number of video consultations per admission, ability to activate a portal account, number of symptom trackers completed and number of messages sent through the portal. Data were extracted *via* a report in the EMR and analysed using descriptive statistics.

Acceptability: Online anonymous surveys were developed for clinicians who were involved in the daily management of patients with COVID-19 infection and parents of children who were admitted to HITH with COVID-19 infection between 7th October 2021 and 28th April 2022. Consumers (patients and families) were asked to rank their satisfaction with the model across 4 domains (sense of safety, impact on stress levels, timeliness of care, overall satisfaction) on a Likert scale of 1–5. Clinicians were asked on a scale of 1–10 how highly they would recommend this model [net promotor score (NPS)]. Both clinicians and consumers were asked to rank each aspect of the model of care (video consultations, remote oximetry, portal messaging, symptom tracking) in terms of usefulness on a Likert Scale of 1–5. Free-text comments were themed by two researchers, one involved in the study (JL) and the other an independent improvement manager to avoid unintentional bias. The survey was distributed *via* email to clinicians and through the portal to consumers. The portal was chosen for the consumer survey as this would target those who were able to engage with all modalities of the virtual model.

Safety: Emergency presentations to hospital during the admission, escalation in care to the ward or intensive care were extracted from the EMR.

Impact: Total number of patients with COVID-19 infection managed through the virtual model on HITH and number of bed-days that would otherwise have been in hospital. Each patient managed virtually accounted for 1 nursing visit that was avoided. Nursing ratios were taken from the daily roster over the seven month period. Nursing travel time and petrol saved were calculated using patient postcodes extracted from the EMR and the beeline distance to the Royal Children's Hospital postcode (3052) to determine approximate distance from hospital and travel saved. Calculations were based on average fuel consumption published by Australian Bureau of Statistics (16) and average retail petrol prices published by Australian Competition & Consumer Commission (17).

Ethics approval for quality assurance project was obtained from the RCH Ethics Department (QA/90835/RCHM-2022).

## Results

Between 7th October 2021 and 28 April 2022 there were a total of 331 unique patients admitted a total of 354 times (23 repeat admissions) to RCH HITH with COVID-19 infection. Of the 23 who were readmitted, the median time between discharge and readmission was 4 days (range 1–82 days). 91% (*n* = 21) were readmitted within 8 days which likely represents worsening symptoms in the same illness. Two patients (8.7%) were readmitted months later, likely representing repeat infection.

Feasibility: All patients (*n* = 331) successfully engaged in video consultations with up to 5 virtual assessments per day (average 1.3 per patient per day). 192/331 (58%) successfully signed up to the EMR patient-facing portal and completed the symptom tracking questionnaire 634 times (average 3.3 per patient) and 783 portal messages (average 4 per patient). All patients (*n* = 331) were given an oximeter to use at home, and all were able to obtain appropriate readings either supported virtually through a video consultation or independently through the symptom tracker.

**Figure 2:**
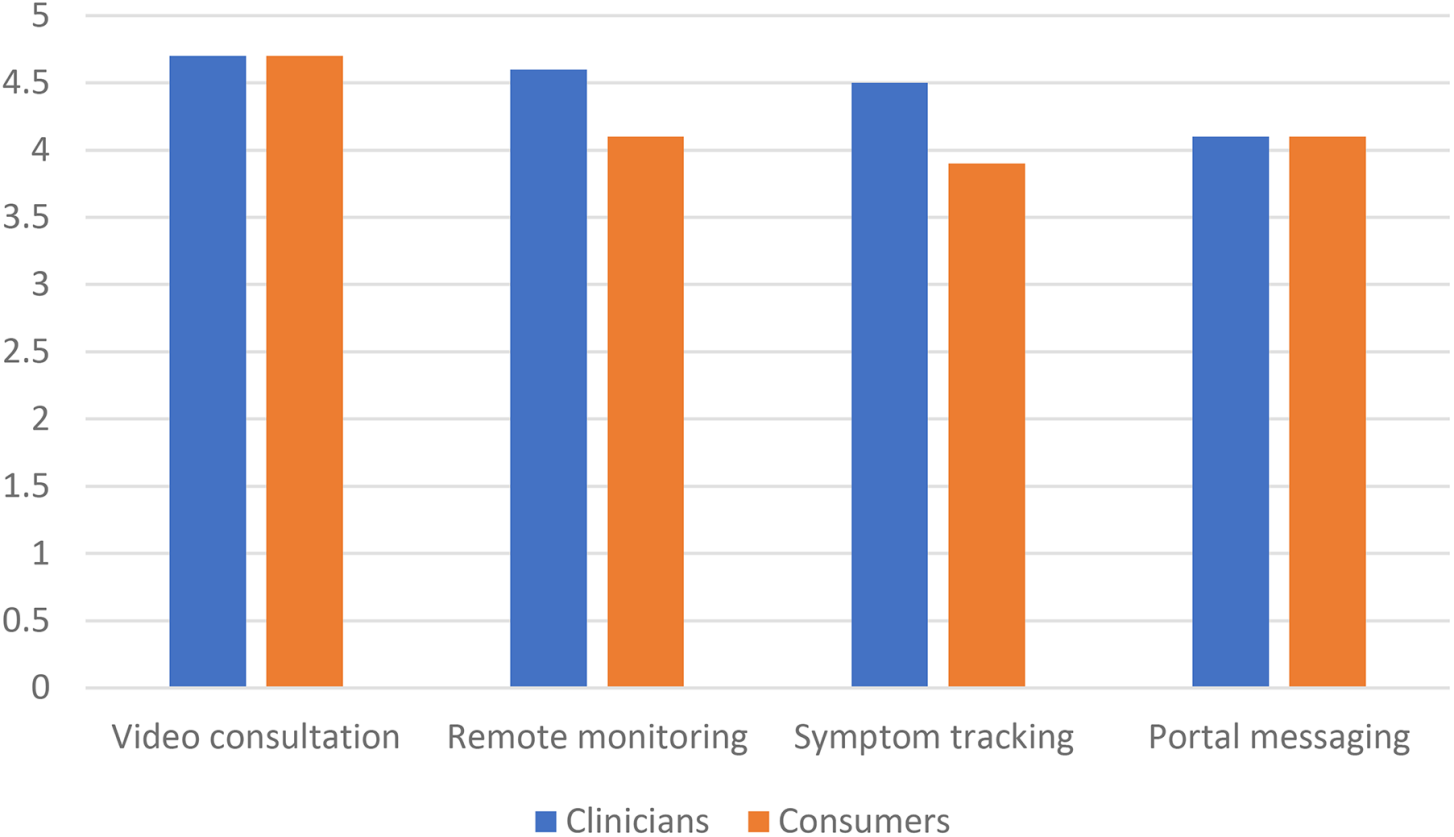
Most useful aspects of the model (with average rating out of 5).

Acceptability: There were 30/168 (18%) consumer responses with overall mean satisfaction 4.7 on a 5 point Likert scale (range 3–5) (Figure 2). Free text comments (*n* = 7) all captured gratitude for being cared for at home and the impact on stress levels: “*very grateful that someone kept an eagle eye on my son as it was a very stressful time given his other health issues”* and “*my son’s stress levels were significantly reduced being in his own home environment and knowing RCH was a video call away and he was being monitored remotely”*.

*“The families that filled in the symptom tracker and arrived at their appointments on time made the program work really well. The ones we had to call and chase up wasted a lot of time and resources.”*

“This system works well for health literate English speaking parents but the ones who perhaps need this service more (less child care/ supports etc) it does not work as well”

Video consultation was rated the most useful aspect of the program by both clinicians (4.7/5, range 2–5) and consumers (4.8/5, range 4–5), followed by remote monitoring. Consumers rated symptom tracking questionnaire as being more useful than portal messaging, however clinicians rated the portal messaging above the symptom tracking questionnaire (Figure 1). The largest difference in rating between clinicians and consumers was for the symptom tracking questionnaire, which consumers found more useful (4.5) than clinicians (3.9). This difference was non-significant (*p* = 0.37), acknowledging small sample sizes.

Safety: There were no significant adverse events at home during the 7-month period. There were 8 children who represented to hospital (2.4%) with 7 being re-admitted (2.1%). There were no admissions to intensive care directly from HITH.

Impact: Average length of stay was 3.5 days per patient with a total of 1,312 bed-days saved across the 354 admissions. In addition, the virtual nature of this model, means 1,480 in-home visits were avoided. This equates to 2,493 hours on Australian roads spanning 52,888 kms, 6,311 kg of CO_2_ and $A5,194 in petrol costs, assuming travel to and from RCH from our traditional model (Figure 1). Due to the lack of travel time between patients, nursing ratios for the virtual model were ten patients per nurse compared to on-road nursing ratios of five patients per nurse.

## Discussion

A virtual HITH model to care for paediatric patients with COVID-19 infection in their own homes is feasible, safe and acceptable to families and clinicians. The impact of such a model saves a substantial number of bed-days compared to hospitalisation and saves nursing travel time compared to traditional in-home visiting. The latter allowed for this time to be spent by nurses assessing more patients and occurred with very low readmission rates.

The most useful aspect of the model was considered the video consultation by both consumers and clinicians. Video consultations have risen dramatically during the pandemic with many ambulatory settings converting to video consultation to support the “stay at home” campaigns and reduce infection risk. Paediatric studies in our local context have shown video consultation to be associated with a positive parent experience in the ambulatory context (5, 14). Less has been published in the inpatient context, and none for HITH. The ability to connect audio-visually with a clinician to discuss concerns directly highlights the usefulness of real time discussion in the care delivery for acutely unwell patients. The ability for the clinician to perform a visual assessment of the patient, augmented by having real-time oximetry data, is also likely to have contributed to the high clinician satisfaction. From a clinician perspective, normal vital signs are likely to have provided reassurance and validation of the clinical assessment. Perhaps for consumers, knowing they were being monitored also provided a sense of reassurance.

While both found it useful, consumers rated the value of the symptom tracking higher than clinicians. It is likely that this tool would provide higher value to clinicians at scale. At the beginning of the pandemic, it was uncertain how many patients would need to be cared for by a static pool of clinicians. Had much higher numbers been seen, the ability to triage and prioritise through a digital tool may have realised much higher value. Video consultations are the most resource-intensive aspect of our model of care, and at higher patient numbers, routine second daily video consultations might have been dropped. The reliance on remote symptom tracking in this scenario would prove very useful. Portal messaging allowing asynchronous communication was a popular aspect of the model. Being able to field non-urgent queries without both parties needing to be available at the same time holds benefits for both consumers and busy clinicians.

Consumer satisfaction sought through the portal limits our pool of consumers to those who were able to engage in all aspects of the model of care. This excludes patients who were unable to create a portal account due to difficulties with English or concerns about child vulnerability. With a low response rate of 18%, this is not a broad representation. At best we can conclude that this model of care was popular for a subset of patients. As models of virtual care increase around the globe, digital health equity has gained increasing focus. To ensure those who are unable to engage with technology are not left behind, in-person models of care cannot be completely forgone. In addition, developing functionality of the technology for those whose first language is other than English, should be a priority for digital developers.

This study has some limitations. Whilst we can conclude patients will engage in this model of care, we are unable to tell what uptake would have been if a choice had been offered between virtual and in person care. There is a growing awareness that consumers want choice in their care delivery and whilst virtual would have suited many families, others may have felt more reassured with in-home visits (15). Likewise, we conclude that this model is safe based on no adverse events over a 7-month period, when patient numbers being cared for through the virtual model reached a maximum of 15 per day. Because our stringent entry criteria kept the numbers of daily patients relatively controlled, this limits the ability to assess how the model would work at scale. However, it might be concluded that having broader criteria to care for children with milder illness, as long as the service was resourced to do this, would be similarly safe and acceptable.

Given this new virtual model of care was implemented out of necessity and in rapid response to an escalating COVID scenario, the authors were limited in their ability to control inherent biases. Despite this, what this retrospective study has shown is the ability for clinicians to quickly and collectively adapt and pivot from the traditional face to face model of care towards a more efficient model without drastic changes to existing resources. Key enablers of this include the level of urgency felt by the HITH department given the escalating situation, strong support from HITH leadership to drive this project, regular departmental communication and education to prepare clinicians for the new model and an EMR system that was able to deliver the required functionality without external resources.

This model of care shows promise as an alternative to in-person care. The benefits of being able to manage a large cohort of patients at scale is likely to become increasingly important as our population continues to grow and hospital demand outstrips supply. The environmental benefits of reduced travel, as we navigate a world challenged by climate change, should also not be overlooked. A cost-effectiveness study would likely add further weight to the argument to shift towards virtual care models. The costs of this model of care with higher patient ratios per nurse, no travel costs and substantially reduced hospital overheads would make this a significantly more cost-effective method to look after patients requiring hospital-level care. The implications for children with illnesses caused by other respiratory viruses and transforming the way hospital care is delivered are enormous.

## Data Availability

The raw data supporting the conclusions of this article will be made available by the authors, without undue reservation.
